# Six-Degree-of-Freedom Posture Measurement Technologies Using Position Sensitive Detectors (PSDs): State of the Art

**DOI:** 10.3390/mi13111903

**Published:** 2022-11-03

**Authors:** Xiangxu Meng, Siwei Sun, Xuetao Yan, Fengman Liu, Liqiang Cao, Qidong Wang, Yu Sun

**Affiliations:** 1 System Packaging and Integration Research Center, Institute of Microelectronics, Chinese Academy of Sciences, Beijing 100029, China; 2 School of Microelectronics, University of Chinese Academy of Sciences, Beijing 100049, China

**Keywords:** 6-DOF posture measurement, position sensitive detector, large-scale equipment assembly, ultra-precision manufacturing

## Abstract

Six degree-of-freedom (6-DOF) posture measurement is an important academic research topic which has been broadly applied in many fields. As a high-speed photoelectronic sensor with ultra-high resolution and precision, position sensitive detector (PSD) has shown to be one of the most competitive candidates in 6-DOF measurement. This review presents the research progress of PSD-based 6-DOF posture measurement systems in the field of large-scale equipment assembly, ultra-precision manufacturing and other emerging areas. A total of six methods for implementing 6-DOF measurement are summarized and their advantages and limitations are discussed. Meanwhile, the paper illustrates challenges, potential solutions and future development trends.

## 1. Introduction

Six-degree-of-freedom (6-DOF) posture of an object refers to its 3D translation and 3D rotation along the x, y, z axes, which is a complete description of its motion state [1,2]. Six-DOF measurement technology, which is proven to be a hot topic in both industry and academia, involves diverse sensory techniques, system configurations and error compensation algorithms. A very broad range of fields need 6-DOF posture measurement and their measurement requirements are quite diversified, as shown in Table 1 [3,4,5,6,7]. 

In order to provide the reader with an intuitive understanding of the principles of 6-DOF measurement, Table 2 lists four mainstream 6-DOF measurement technologies with a brief analysis of their advantages and disadvantages. Compared to inertial methods and GPS navigation methods, optical methods have outstanding advantages of high accuracy, immune to electromagnetic disturbances and contactless measurement. The last few decades have observed a remarkable research effort directed towards optical 6-DOF posture measurement methods. Herein, we mainly focus on the 6-DOF measurement using optical methods. After comprehensive investigations, the challenges faced by traditional 6-DOF measurement technologies are summarized as follows.

- Difficult to achieve ultra-high precision, better than ± 1 μm and ± 1;
- Difficult to achieve measurement speed above 5 kHz;
- Hard to measure the 6-DOF posture simultaneously with simple configuration;
- Hard to measure the roll angle precisely;
- Lack of miniature systems capable of working in tiny spaces.

To overcome the challenges mentioned above, it is crucial to select appropriate sensors, as sensors play a critical role to provide signals related to the target movement and determine the performance limits of the integrated system. Position sensitive detector (PSD) is particularly well-suited for measuring posture due to its outstanding accuracy, excellent resolution, superior speed and simple signal processing [13,14,15]. To date, PSD-based 6-DOF posture measurement systems have been extensively studied and much valuable work has been reported.

The research work on the 6-DOF measurement is mainly focused on two applications: high-end equipment assembly and ultra-precision manufacturing. High-end equipment refers to major infrastructure with extra-large size such as aircrafts, submarines, high energy particle accelerators and so on. During their assembly process, 6-DOF posture measurement is essential to control the assembly accuracy and guarantee the equipment quality. The main large-scale pose measurement technologies include laser tracking, indoor GPS and digital photography [16,17,18]. 

**Table 2:** Six-DOF measurement technologies.

Technology	Principle	Accuracy	Speed	Advantages	Limitations
Laser Tracking [19] 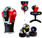	Spherical coordinate system; Laser interference ranging; Angle encoders.	15 μm + 6 μm/m	3000 Hz	(1) High precision; (2) High speed; (3) Portable & flexible.	(1) Station-transfer calculation; (2) Contact measurement.
Indoor GPS [20,21] 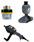	Using multiple emitters; Angular velocity × time = azimuth; Triangulation principle.	0.12 mm @ 10 m	20 Hz	(1) Parallel measurements; (2) Large range.	(1) Low speed; (2) Low accuracy; (3) Contact measurement.
Digital Photography [22] 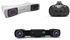	Perspective N points (PnP); Two CCD cameras; Image processing.	4 μm + 4 μm/m	10 Hz	(1) High precision; (2) Non-contact; (3) Simple hardware.	(1) Low speed; (2) Sensitive to variations.
Inertial measurement unit [23] 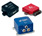	Accelerometers + gyroscopes; Acceleration × time^2^ = translation; Angular rate × time = orientation.	10 mm/s	3600 Hz	(1) High speed; (2) Light weight.	(1) Error accumulation; (2) Contact measurement; (3) Multiple error sources.

Among these three technologies, laser tracking and digital photography are superior to indoor GPS in terms of measurement accuracy, and these two technologies both utilize PSDs as key elements. In the area of ultra-precision manufacturing, 6-DOF measurement is intended for calibrating the geometric motion error of the linear guide. The most representative instrument for accurate calibration is laser interferometer [24]. However, the laser interferometer has three major disadvantages. Firstly, any variations in the testing environment that affect the laser wavelength (such as temperature, pressure and humidity, etc.) will seriously reduce the measurement accuracy. Secondly, the laser interferometer cannot self-recover from interruptions (such as beam blockage, loss of light, etc.), causing difficulties in measuring over long time periods [25]. Lastly, the system will be rather complex when using laser interferometers to implement simultaneous measurement of 6-DOF motion [26,27]. In contrast, PSDs are insensitive to pressure or humidity variations and capable of detecting discontinuous or pulsed light, thus are very suitable for calibrating the linear stage with high efficiency, high stability and easy system configurations. In addition to the two previously mentioned fields, research into 6-DOF measurement also exists in other emerging areas such as space station experiments, indoor positioning and so on. These areas require diversified and customized 6-DOF measurement schemes. By taking PSDs and optics as basic building blocks, various measurement systems can be created to flexibly meet the specific requirements. 

This review retrieved academic papers from 2000 to 2022 using “6-DOF posture measurement” and “Position sensitive detector” as keywords. After thoroughly and carefully eliminating irrelevant search results, there were 98 appropriate papers remaining. By intensively reading these preliminary selections, six main methods for implementing posture measurement are summarized. Then we conducted second screening for each method and retained those high-quality articles with significant works and sufficient details. The reference lists of final selected articles were further checked to avoid omitting any valuable work. Finally, a total of 73 articles were selected in this review.

The rest of this review is organized as follows: Section 2 introduces the main 6-DOF measurement metrics and PSD working principles. Section 3 summarizes the research progress of PSD-based 6-DOF posture measurement in three subsections: Section 3.1 introduces the laser tracking method and binocular method for implementing high dynamic range pose measurement. Section 3.2 introduces the laser diffraction method and laser collimation method for high-resolution and high-accuracy pose measurement. Section 3.3 presents the kinematic analysis method and angle-of-arrival method for compact and flexible pose measurement. Section 4 discusses the advantages, challenges and future directions of PSD-based 6-DOF measurement technology. The last section summarizes the entire paper.

## 2. PSD: A Promising Posture Detector

### 2.1. Performance Indicators of 6-DOF Measurement 

Performance indicators can visually assess the performance of a 6-DOF measurement system and some important indicators are listed below.

1. Resolution. Resolution refers to the minimum detectable changes in translation and rotation, which is directly dependent on the resolution of the used detector.
2. Accuracy. Accuracy refers to the deviation of the measured value from the ground truth, which is generally expressed as RMS error.
3. Repeatability. Repeatability refers to the variation of measurement results when continuously measure a constant posture over a period. It indicates the severity of random error in the system.
4. Dynamic range. Dynamic range refers to the translation and rotation range that the system can properly detect. It should be greater than the motion scope of the target to be measured.
5. Measurement speed. Measurement speed refers to the frequency of updating the measurement results. High measurement speed guarantees the real-time performance of the system.

### 2.2. Working Mechanism of PSD

**Figure 1:**
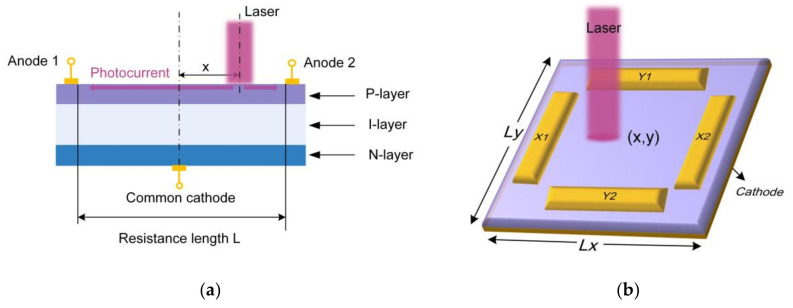
Working mechanism of PSD: (**a**) the cross-section of a one-dimensional PSD; (**b**) schematic diagram of two-dimensional tetra-lateral PSD.

PSD is a monolithic detector without discrete elements, which can directly measure the position of the gravity center of the light intensity based on lateral photoelectric effect [28,29,30]. Figure 1a shows the cross-section of a one-dimensional PSD and explains its operating mechanism. The PSD has a P-I-N structure where the P-layer serves as a uniform resistive layer to distribute the photocurrent and the N-layer is connected to the common electrode to provide reverse bias to the PN junction. A pair of output electrodes are formed on both ends of the P-layer [31]. When a beam is irradiated onto the PSD surface, photocarriers proportional to the light intensity are generated at the incident point. Then a lateral electric field is established in a direction parallel to the PN junction, which drives the photogenerated carriers to drift along the resistive layer towards the electrodes [32,33]. The x-coordinate of the incident position can be solved by the following expression:

(1) $$x=\frac{L}{2}\times \frac{I_{2}-I_{1}}{I_{2}+I_{1}}$$

where $I_{1}$ and $I_{2}$ are the photocurrent output from anode 1 and anode 2, respectively, and L represents the side length of photosensitive area. Similarly, for the two-dimensional tetra-lateral type PSD as shown in Figure 1b, the conversion formula is given by [34]:

(2a) $$x=\frac{L_{x}}{2}\times \frac{I_{X1}-I_{X2}}{I_{X1}+I_{X2}}$$

(2b) $$y=\frac{L_{y}}{2}\times \frac{I_{Y1}-I_{Y2}}{I_{Y1}+I_{Y2}}$$

where $I_{X1}$, $I_{X2}$, $I_{Y1}$, $I_{Y2}$ are the photocurrent output from the corresponding anodes, and $L_{x}$, $L_{y}$ represent the length of photosensitive area in the *x*-direction and *y*-direction, respectively.

### 2.3. How Does PSD Solve the Challenges

PSD is one of the most competitive and promising detectors in 6-DOF posture measurement. It can directly detect the two-dimensional position of the incident beam, from which the 6-DOF full posture can be derived. PSDs offer new pathways to solve a range of challenges faced in the 6-DOF posture measurement systems.

(1) PSD significantly improves the measurement accuracy.

The Lucovsky equation has mathematically proven that the photocurrents output from an ideal PSD structure are perfectly linear to the light incident position, which means that PSD is highly precise in position detecting [35]. In fact, a central photosensitive area with a diameter of 100 μm is sufficient for ultra-precise posture measurements, where the PSD accuracy can reach the sub-micron level [36,37].

(2) PSD significantly improves the measurement resolution.

The photosensitive surface of PSD is continuous so that it can provide unsurpassed analog resolution. The spatial resolution of PSD can reach less than 0.1 μm, which can fully satisfy the resolution requirements in most applications [38].

(3) PSD enables ultra-fast measurement.

The response time of PSD includes carrier transition time and the RC time constant and has a typical value of approximately 1 μs [39]. In addition, the signal processing of PSD is rather simple (refer to Equation (2)) and time-saving. The measurement speed of a PSD-based system can easily exceed 1 kHz, which helps to improve measurement efficiency, shorten measurement time and provide capability of measuring high-speed dynamic targets.

(4) PSD greatly simplifies the system configuration.

In theory, 6-DOF measurement can be realized with just three PSDs orthogonal to one another [10], and the system can be further simplified by adopting frequency modulation, as a single PSD can detect multiple incident light modulated at different frequencies simultaneously [40]. Therefore, the PSD-based systems can be highly integrated with much reduced sizes and are capable of working in tiny spaces with the advantages of flexibility and portability. 

Overall, PSDs have shown great application value in 6-DOF measurement and can open new possibilities for overcoming a series of performance bottlenecks.

## 3. 6-DOF Posture Measurement Systems Using PSDs

This section reviews the research advances of 6-DOF posture measurement systems using PSDs in high-end equipment assembly, ultra-precision machining and other emerging areas.

### 3.1. Large-Scale 6-DOF Posture Measurement in Equipment Assembly

#### 3.1.1. Laser Tracking Technology

**Figure 2:**
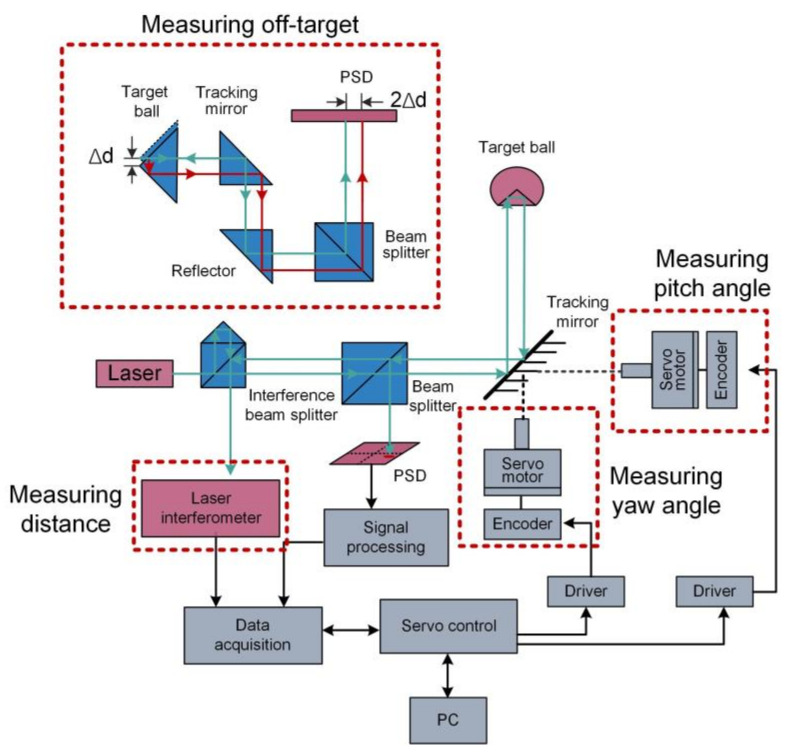
System diagram of a laser tracker and the principle of measuring the miss distance.

The laser tracking technology provides real-time tracking and high-precision polar coordinates measurement of dynamic targets and its system diagram is shown in Figure 2. Miss distance are usually measured by quadrant detector (QD) or PSD is used to measure the miss distance quantity to track the target while the laser interferometer together with two angle encoders are used to measure the polar coordinates of the target. 

In the early work, researchers used four-quadrant detectors (QD) to measure the miss distance, and the measurement accuracy of miss target was 5 μm within ±500 μm measuring range [41]. However, research into measuring the miss distance with QDs face the following problems [42,43]:

(1) The measurement results are relevant to spot radius. QD estimates the spot position based on the distribution of the light intensity in each quadrant, and the relationship between the QD reading and the actual spot displacement is expressed as:
  (3) $$d_{x}\approx \frac{2x_{0}}{\pi r},\;\;\;d_{y}\approx \frac{2y_{0}}{\pi r}\;\;\;\;\left(\left|x_{0}\ll r\right|,\;\left|y_{0}\ll r\right|\right)$$
  where r is the radius of light spot, $\left(d_{x},\;d_{y}\right)$ represents the QD reading, and $\left(x_{0},\;y_{0}\right)$ is the actual spot displacement. The content in brackets indicates that Equation (3) is valid on the condition that the coordinates of the spot should be much smaller than its diameter. From Equation (3), it is obvious that the QD reading is non-linear with the change of spot radius.
(2) The measurement results are relevant to spot modes. QD shows the highest response for Airy spots but the lowest for uniform spots.
(3) The measurement range is limited. Since the light spot must cover all quadrants of QD during the detection, which directly limits the measurement range and leads a narrow field-of-view (FOV).

In recent years, many researchers have turned to PSD for miss distance measurement. The detection results of PSD are independent of spot size and spot mode because it directly detects the center of gravity of spot intensity. Additionally, the FOV of PSD is wider than QD since PSD can achieve continuous detection of the spot over the entire photosensitive surface. 

**Figure 3:**
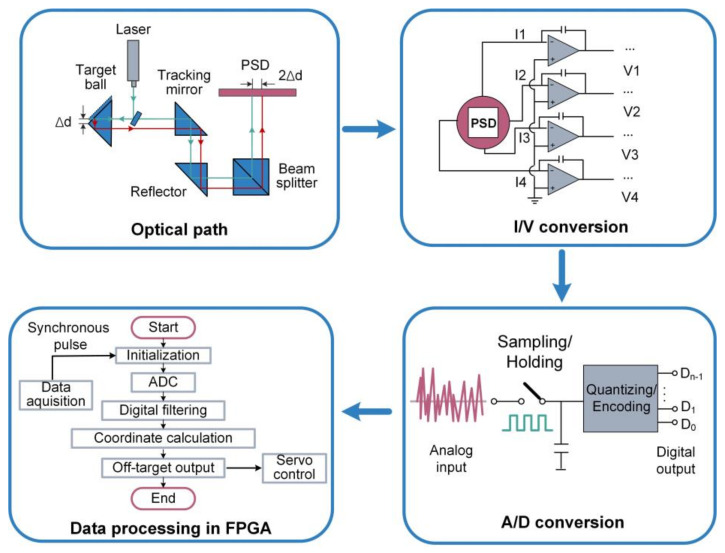
Block diagram of miss distance measurement system.

Dong et al. proposed a high-precision miss distance measurement system using a PSD (SiTek, 2L10_SU7) to replace QD [44,45]. The block diagram of system is shown in Figure 3, which mainly includes a front-end optical path, PSD and its signal processing circuit and a back-end data processing module. In order to achieve precise and stable miss distance measurement, the system is carefully designed in both hardware and software. In terms of hardware design, a filter capacitor network is developed to suppress the power ripple. The input of the operational amplifier is separated from a large voltage on the PCB to ensure the weak photocurrent can be detected. In terms of software design, arithmetic average filtering and median filtering are used to reduce the noise of sampled data. The PSD-based miss distance measurement system showed a repeatability accuracy with greater than ±2 μm within an area of 4 × 4 mm^2^, which is better than QD-based system in terms of both accuracy and measurement range.

#### 3.1.2. Digital Photography Technology

Digital photography technology is another high-precision posture measurement method in large equipment assembly, which can be divided into monocular vison and binocular vision. Monocular vison can only measure the two-dimensional information of the target, which limits its application. By contrast, binocular vision can perform 3D measurement with simple structure and large measurement range, making it become the major method in digital photography technology [46,47]. Charge coupled device (CCD) has been widely adopted in binocular vision systems, but it is not possible to be both accurate and fast, and is also vulnerable to the change of ambient light [48]. PSD is expected to be more appropriate in some binocular systems since it can operate at extremely high speed without deteriorating accuracy. In addition, PSD-based binocular systems can get rid of the complex image processing tasks and enhance the measurement stability.

**Figure 4:**
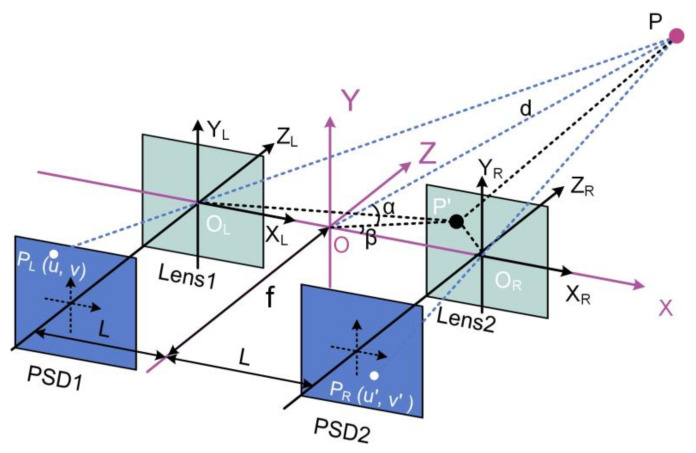
Binocular vison system based on dual PSD cameras.

Based on dual PSD cameras (PSD + lens), Liu et al. developed a binocular visual measurement system as shown in Figure 4 [49]. Two PSDs are fixed in the focal plane of the lens with focal length *f* and separated by distance 2L. The world coordinate system O−XYZ is established by taking the midpoint of the baseline as the origin. By geometric analysis, the polar coordinates of point $P\left(d,\alpha ,\beta \right)$ can be expressed as:

(4a) $$d=\frac{L\sqrt{u^{2}v^{\prime 2}-2uu^{\prime }vv^{\prime }+u^{\prime 2}v^{2}+4v^{2}v^{\prime 2}+4v^{\prime 2}f}}{uv^{\prime 2}+u^{\prime }v}$$

(4b) $$\alpha =\arcsin\left(\frac{2v^{\prime }f}{\sqrt{u^{2}v^{\prime 2}-2uu^{\prime }vv^{\prime }+u^{\prime 2}v^{2}+4v^{2}v^{\prime 2}+4v^{\prime 2}f}}\right)$$

(4c) $$\beta =\arcsin\left(\frac{2vv^{\prime }}{\sqrt{u^{2}v^{\prime 2}-2uu^{\prime }vv^{\prime }+u^{\prime 2}v^{2}+4v^{2}v^{\prime 2}}}\right),$$

where $\left(u,v\right)$ and $\left(u^{\prime },v^{\prime }\right)$ are readings of PSD1 and PSD2, respectively. To verify the proposed measurement model, a series of experiments were carried out using a 340 × 340 mm^2^ panel as the target. The target motion space was 160 × 160 × 200 mm^3^ with depth distance of 1000 mm away from dual-PSDs. The experimental results showed that the mean positioning error is 4.35 mm.

The proposed binocular vision measurement model is based on the principle of optical triangulation with simple algorithms and fast operation. However, the various non-linearities during actual operation are not considered, such as radial and tangential aberrations in the lens, pincushion or barrel distortions of PSD and inconsistencies in different channels of circuit. A more advanced measurement model is needed to incorporate the various non-linear factors and further improve the measurement accuracy.

**Figure 5:**
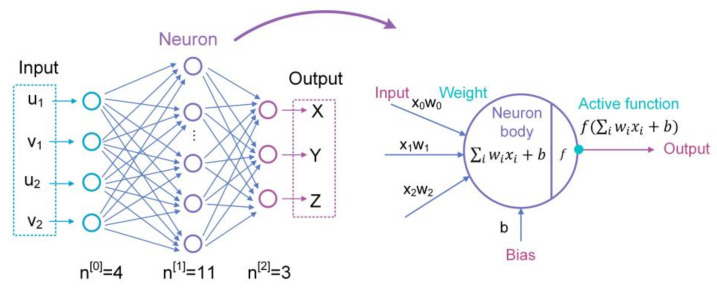
Structure of the constructed BP neural network.

A back propagation (BP) neural network, which is skilled in dealing with systems that are difficult to describe with an accurate mathematical model, has been widely incorporated into the binocular vison systems to deal with non-linearity factors [50,51,52]. Wang et al. built a high precision binocular vision localization system using a back propagation (BP) neural network [53]. As shown in Figure 5, the dual-PSD readings $\left(u_{1},v_{1}\right)$ and $\left(u_{2},\;v_{2}\right)$ are used as the input values, then the target coordinates $\left(X,Y,Z\right)$ can be obtained from the output of the BP neural network. Genetic algorithm (GA) is also used to provide optimized initial parameters as the starting point of BP iteration process. The final BP neural network has a three-layer network structure with a single hidden layer consisting of 11 neurons. After being trained by Levenberg-Marquardt optimization method, the BP neural network can measure the target with an accuracy of 1.66 mm within motion space range to 300 × 300 × 200 mm^3^.

The BP neural network is effective in improving the positioning accuracy, but it is prone to fall into local optimum solutions. A number of samples are necessary in the training process, which may expose the system with excessive training time. It is speculated that if the 3D coordinates are estimated roughly by a physical imaging model, and then use BP neural network to correct the residual non-linear errors, the measurement accuracy will be further improved to sub-millimeter levels.

**Figure 6:**
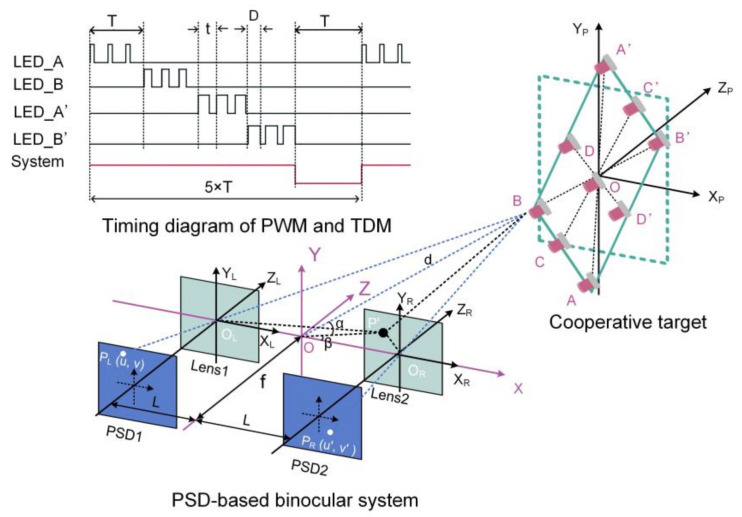
6-DOF full posture measurement using the cooperative target.

In order to complete the measurement of 3D rotation after the 3D translation is obtained, the PSD-based binocular vision system needs a cooperative target which consists of multiple light sources of known arrangement. Cheng et al. demonstrated a 3D rotation measurement model as shown in Figure 6 [54]. The cooperative target has nine LEDs, the light intensity of which were pulse-width modulated (PWM) and time division multiplexed (TDM). AA’BB’ and CC’DD’ are two sets of orthogonal and independent feature beacons, and they are used as the primary solution and auxiliary solution, respectively. The final result is obtained by averaging the primary solution and the auxiliary solution. Firstly, the 3D positions of each LED were firstly measured to construct a set of vectors, and then the final 3D rotation is described by the angle between specific vectors. Taking the first set of beacons as an example, the 3D rotation is calculated as:

(5a) $$\theta =\frac{\pi }{2}-\arccos\left(\frac{\lambda \cdot i}{\left|\lambda \right|\left|i\right|}\right)$$

(5b) $$\psi =\arccos\left(\frac{\lambda^{\prime }\cdot k}{\left|\lambda^{\prime }\right|\left|k\right|}\right)$$

(5c) $$\varphi =\arccos\left(\frac{\omega \cdot j}{\left|\omega \right|\left|j\right|}\right),$$

where $i=\left(1,0,0\right)$, $j=\left(0,1,0\right)$, $k=\left(0,0,1\right)$, $\;\lambda =\frac{AA^{\prime }}{\left|AA^{\prime }\right|}\times \frac{BB^{\prime }}{\left|BB^{\prime }\right|}$ and ω is the projection of vector $\frac{BB^{\prime }}{\left|BB^{\prime }\right|}$ on the $O_{P}-X_{P}Y_{P}$ plane. Due to equipment limitations, only pitch and yaw angles were tested. The experimental results showed that the measurement errors for pitch and yaw angles were 0.923° and 0.563° within a dynamic range of ±15°, respectively.

Overall, the PSD-based binocular vision systems can directly measure the 3D position but the 3D orientation is indirectly measured by extrapolating the position distribution of different LEDs on the cooperative target as PSDs cannot capture pictures like CCDs, which means PSD-based binocular vision systems cannot tackle noncooperative targets. In fact, the measurement accuracy of non-cooperative targets is not so high as cooperative targets, especially when the background is complex and the target surface lacks texture. Once beacons on the target are designed as feature points, PSD-based binocular systems are more preferred than CCD-based systems for its rapid, precise and stable performance.

### 3.2. Ultra-Precision 6-DOF Motion Error Measurement of Linear Stage

#### 3.2.1. Laser Diffraction Method

The laser diffraction method utilizes a diffraction grating to achieve high-resolution and high-precision motion measurement of linear stage. According to the grating equation, the directions of the diffracted rays are influenced by the incident angle [55]. Thus taking the diffraction grating as the target to be measured, the 6-DOF motion of the linear stage can be deduced from the origin and direction of the diffracted rays.

**Figure 7:**
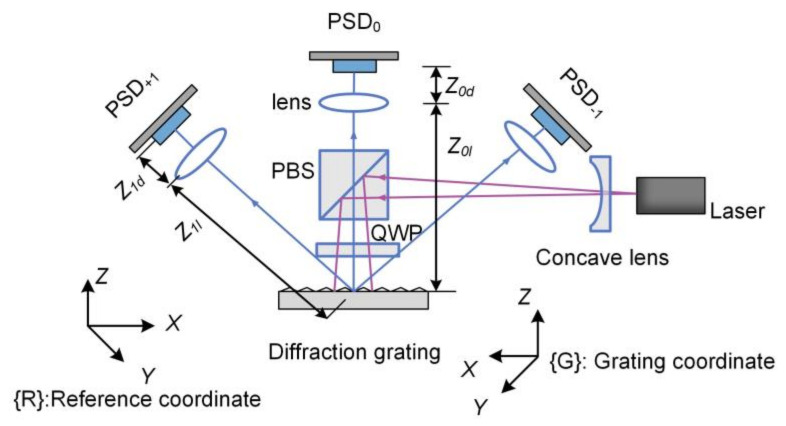
6-DOF motion measurement system using laser diffraction method.

Kim et al. proposed a 6-DOF posture measurement system using a circular diffraction grating [56]. The system is symmetrical as shown in Figure 7. The ray emitted from the laser source is vertically incident on the diffraction grating. The diffraction grating generates +1, 0, and −1-order diffracted rays, which then reach on the surfaces of three PSDs. $Z_{0l}$ and $Z_{1l}$ represent the distance from the lenses to the diffraction grating of 0 and +1 order rays. $Z_{0d}$ and $Z_{1d}$ represent the distance from the PSDs to the lenses of 0 and +1 order rays. The design parameters of −1 order ray have the same values with +1 order ray due to symmetry. The 6-DOF posture of the diffraction grating is calculated by kinematic analysis, and the coordinate transformation matrix TGR is derived from the coordinates of the diffracted rays on the PSDs using a numerical iterative algorithm. The experiment verified that the translation accuracy was ±10 μm and the rotation accuracy was ±0.012°. The repeatability of translation and rotation were 10 μm and 0.01°, respectively. The translation measurement range was ±1 mm, and the measurement range of roll, pitch and yaw were ±0.54°, ±0.39°, and ±1.64°, respectively.

In the subsequent research, the authors proposed a new design methodology to optimize the system [57]. A complex performance index (CPI) is defined to consider multiple performance parameters simultaneously. By optimizing CPI, the optimal values of four key parameters ($Z_{0l},\;Z_{1l},\;Z_{0d},\;Z_{1d}$) are determined. Powell’s method and the quadratic interpolation method are employed to obtain the optimal solution. The parameters were finally optimized to be (136.4 mm, 85 mm, 251.6 mm, 170 mm), and the measurement accuracy was significantly improved to ±0.5 μm and ±2″.

The limitation of this system is that it lacks optical analysis, only based on kinematic analysis. The divergence of the light beam was ignored, which results the deterioration of the experimental results from the theoretical analysis values. 

**Figure 8:**
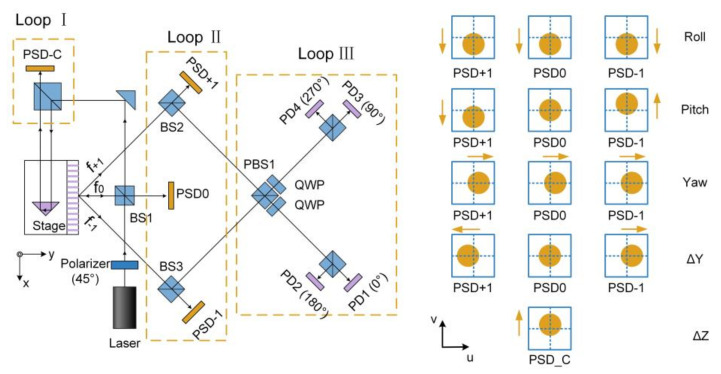
The high-resolution optical encoder for 6-DOF motion measurement.

By adding comprehensive optical analysis, Lee et al. constructed a high-resolution optical encoder for 6-DOF motion measurement applying for an ultraprecision linear stage [58]. The proposed system consists of four PSDs, four photodiodes (PDs), a diffractive linear grating and some auxiliary optics components. The overall system configuration can be divided into three loops: Loop Ⅰ measures ΔZ using PSD_C; Loop Ⅱ measures ΔY, pitch, yaw and roll by three separate PSDs together with a diffractive linear grating; Loop Ⅲ measures ΔX by four PDs. The linear stage motion brought about changes of beam positions on the four PSDs are illustrated in Figure 8. Kinematic analysis is used to decouple the 5-DOF motion, namely, ΔY, ΔZ, pitch, yaw and roll, from the respective beam changes on the four PSDs. The measurement of ΔX is based on the circularly polarizing interferometric technique (CPIT) in a robust manner. In the experiments, the resolution of PSD was evaluated to be better than 0.03″ in rotation and 20 nm in translation. The achieved resolution of the proposed optical encoder was 0.03″ for pitch, yaw and roll, 0.4 nm for ΔX and 20 nm for ΔY and ΔZ.

Above all, the laser diffraction method can achieve high-resolution, high-accuracy measurement of the linear stage motion. However, this method has a limited measurement range due to the scattering of diffracted light. In addition, diffraction gratings are sensitive to the environment so that this method requires a high level of environmental stability.

#### 3.2.2. Laser Collimation Method

**Figure 9:**
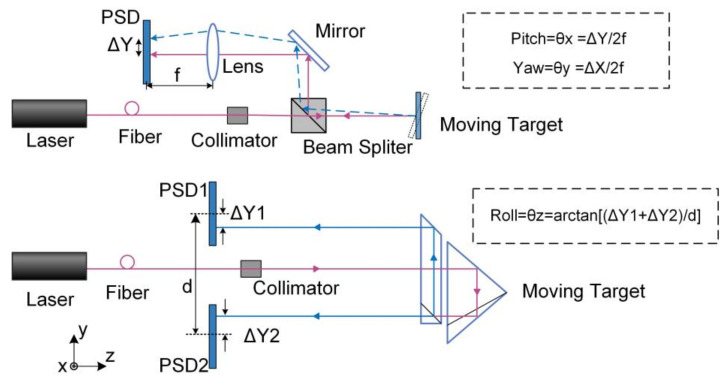
Strategies of measuring pitch, yaw, roll, ΔX and ΔY based on laser collimation method.

The laser collimation method has the advantages of long working distance and high measurement accuracy since the collimated laser has excellent directionality [59]. A detailed description of the pitch, yaw and roll measurement strategy utilizing collimated laser is shown in Figure 9. Pitch and yaw angles are obtained simultaneously by a single PSD. Roll angle is measured using parallel beams incident on two separated PSDs, one of which can be used to measure ΔX and ΔY motion. The ΔZ motion is usually measured by a laser interferometer.

**Figure 10:**
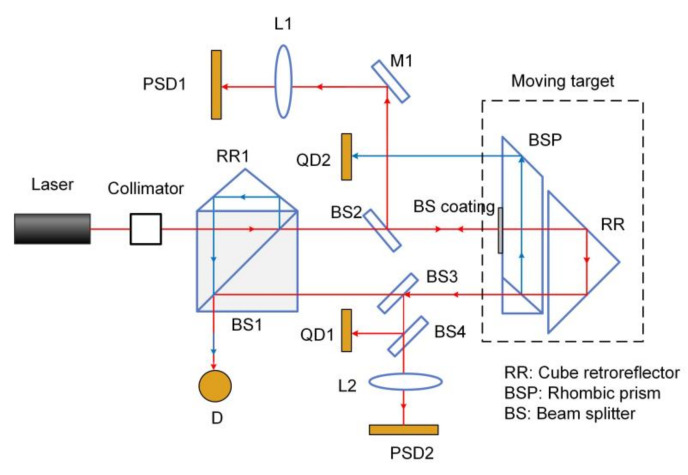
Schematic of the 6-DOF measurement system based on laser collimation method.

Based on laser collimation method, Feng et al. developed a novel system for measuring 6-DOF motion errors of linear stage using a mono-frequency laser interferometer [60]. The schematic of the system is shown in Figure 10, which adopts a rhombic prism (BSP) and a cube retroreflector (RR) as the measured target. The distance is measured by the detector D based on laser interference method. Roll angle, ΔX and ΔY are detected by the combination of QD1 and QD2. Pitch and yaw are detected by PSD1 based on laser collimation method. Laser beam drift is measured by PSD2 based on the principle of common-path compensation. The experiments verified that the resolution is 0.2 μm and 0.25″ for translation and rotation, respectively. The accuracy is 0.8 μm for ΔX and ΔY in the measurement range of ±100 μm. The accuracy of pitch, yaw and roll are 1.3″, 1.0″ and 2.0″ in the measurement range of ±100″, respectively. 

The advantage of the proposed system is the real-time error compensation, since the common-path compensation is embedded directly into the optical path without involving complex computations. The disadvantage is the lack of considering a series of systematic errors, such as fabrication and installation errors of optical elements, parallelism errors of the beam that reaches QDs and the crosstalk between geometric motion errors.

**Figure 11:**
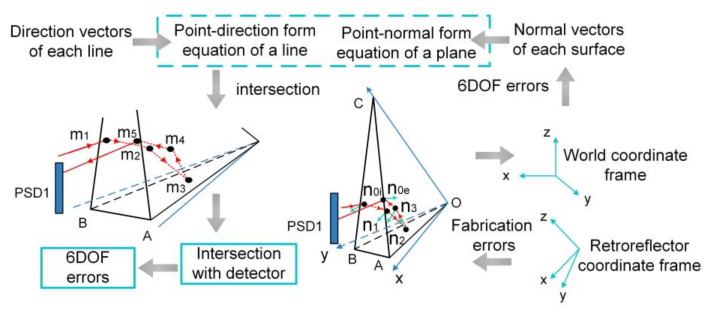
Basic idea of establishing the error compensation model using ray-tracing.

To reduce the systematic errors, Zhao et al. established a measurement model to provide error compensation for ΔX, ΔY, pitch, yaw, and roll motion based on the ray-tracing method [61]. The ray-tracing method simulates reflection and transmission of a ray as it interacts with different optical elements. As shown in Figure 11, the solving process is to use the point-direction form equation of a line and the point-normal form equation of a plane to calculate the intersection of the line and the plane. Once the intersection of the ray and detector has been solved, the measurement model for 5-DOF error compensation can be obtained through mathematical transformation. By applying the proposed model, the measurement accuracy of ΔX and ΔY were verified to be 1.5 μm and 1.16 μm, respectively. The measurement accuracy of pitch and yaw were both improved to 0.89″, and the accuracy of roll was improved to 1.11″. The repeatability for translation and rotation are better than 1 μm and 1″, respectively. The measurement range, in which the linear fitting coefficient is better than 0.9999, is ±150 μm for ΔX and ΔY, ±200″ for pitch and yaw, and ±100″ for roll, respectively. 

The proposed error compensation model is accurate and stable without sacrificing the measurement range. Compared to Feng’s experimental results, the measurement accuracy of pitch, yaw and roll were improved by 32%, 11% and 45%, respectively. However, it seemed that the measurement accuracy of ΔX and ΔY had decreased. The possible reason is that the working distance of this experiment was longer than that in the experiment without error compensation (850 mm vs. 500 mm). Therefore, it was no doubt that the proposed error compensation model was effective. Nevertheless, the error compensation model also creates an extra computation burden on the data processing, which affects the real-time performance of the measurement system.

**Figure 12:**
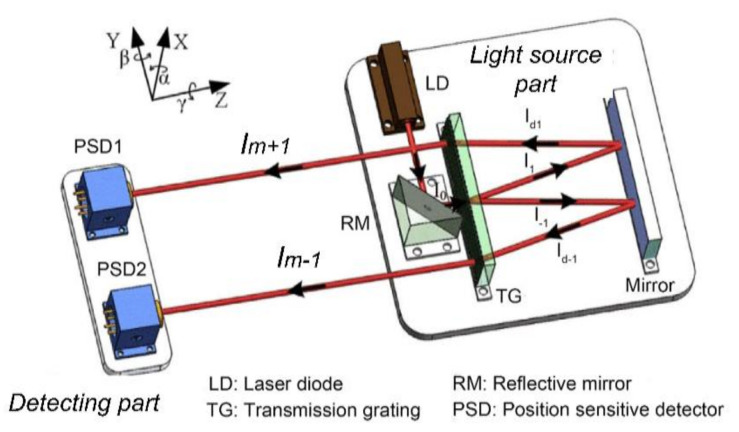
Roll angle measurement system using TG to produce parallel beams.

Hardware-based error compensation is very attractive for its high-speed features. Ren et al. suggested a novel structure for significantly reducing the parallelism error in roll measurement [62]. The parallelism between the two measurement beams is very important, because the parallelism error can greatly reduce the roll measurement accuracy. Moreover, the poor parallelism error can easily cause the spot to overflow the PSD surface, which shortens the working distance of the system. The conventional configuration of generating parallel beams is with a beam splitter together with a mirror, which is sensitive to the fabricating and mounting errors. In contrast, the proposed new method in Figure 12 utilized the ± 1st order diffraction beams produced by a transmission grating (TG), which can theoretically reduce the horizontal parallelism error by 120 times, and almost eliminate the vertical parallelism errors. The actual measurements showed that the horizontal and vertical parallelism errors were 3.15″ and 0.2″, respectively, which was a significant improvement compared to the ~100″ parallelism errors produced by conventional configurations. Finally, the accuracy of the roll angle was tested to be 0.7″ within the range of 70″, and the measurement resolution was 0.5″.

### 3.3. Customized 6-DOF Posture Measurement in Other Emerging Areas

#### 3.3.1. Kinematic Analysis Method

The greatest advantage of the kinematic analysis method is to simplify system structure, which only uses three orthogonal PSDs for simultaneously measuring 6-DOF postures. The basic idea of kinematic analysis is to establish a mapping relationship between PSD readings and 6-DOF postures by performing a coordinate transformation using the homogenous transformation matrix (HTM) [63]. 

**Figure 13:**
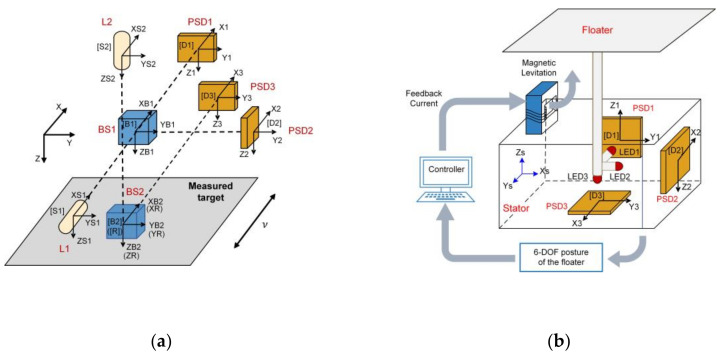
(**a**) 6-DOF geometric motion measurement system for calibrating the mMT; (**b**) 6-DOF posture measurement system used in MAIS.

Based on kinematic analysis method, Wang et al. presented a novel system to calibrate the 6-DOF geometric errors of the miniaturized machine tool (mMT) [64]. The mMT has extremely small dimension thus it requires a highly compact and accurate calibration system. The schematic diagram of the proposed system is shown in Figure 13a, which only included three orthogonal PSDs, two orthogonal laser diodes and two beam splitters. The laser beam emitted by laser diode L1 is received by PSD1 and PSD2, while the laser beam emitted by laser diode L2 is received by PSD3. HTM is used to derive the coordinate relationship between two adjacent measuring components, from which the mapping relationship between the readings of three PSDs and the 6-DOF geometric motion errors of the measured stage can be finally determined. The theoretical resolution of translation and rotation of the proposed system were 0.5 μm and 0.4″, respectively. Experimental results showed that the accuracy of translation in the x, y and z directions were 11.5 μm, 4.5 μm and 3.5 μm, respectively. The accuracy of pitch, yaw and roll angles were 1.65″, 2.06″ and 5.36″, respectively.

With the use of the seven components in total, the proposed system is highly compact, which is ideal for measuring the geometric motion errors of downsized mMTs. Without adjusting the complicated optical path or processing complex error compensation, the proposed system can still achieve high accuracy. The disadvantage of the proposed system is the short working distance of 1.5 mm, which is directly limited by the small photosensitive area (4 × 4 mm^2^) of the PSDs. To improve the working distance or enlarge the measurement range, the use of larger area PSDs is necessary.

Gao et al. developed a more compact 6-DOF posture measurement system, which has been successfully applied in the space microgravity active vibration isolation system (MAIS) of the Tianzhou-1 cargo spacecraft [10,65]. As shown in Figure 13b, the proposed system is further simplified by using three orthogonal laser diodes fixed to the floater and three orthogonal PSDs fixed to the stator, without incorporating any lenses or prisms. Using the coordinate transformation matrix, the 6-DOF motion of the float relative to the stator can be extracted from the PSD readings. According to the experimental results, the measurement accuracy (average deviation) of translation in x, y and z directions were 0.12 mm, 0.15 mm and 0.13mm, respectively. The measurement accuracy (average deviation) of roll, pitch and yaw angles were 0.12°, 0.12° and 0.14°, respectively. The measurement range is better than ±10 mm and ±2.5°.

The realization of large measurement range is attributed to the use of extra-large area PSDs (45 × 45 mm^2^). The disadvantage of the proposed system is the relatively low measurement accuracy, the deterioration of which is partly due to the use of larger PSDs. PSDs with larger areas tend to have more severe dark currents, resulting in heavier non-linearity errors. Therefore, large PSDs with low dark currents are strongly desirable to break the constraint between measurement accuracy and range.

#### 3.3.2. Angle of Arrival Method

The Angle of Arrival (AoA) method is a kind of optical triangulation method, and its counterpart techniques are Time of Flight (ToF) method and Received Signal Strength (RSS) method [66]. The AoA method measures the angular direction (azimuth and elevation) of the signal arriving at a receiver to localize the target in 3D space. The AoA method is relatively easy to implement because it does not require clock synchronization which is necessary for TOF. Moreover, it is not sensitive to power variation, ambient light and multipath effect, which are the typical challenges faced by RSS [67,68]. In recent years, the AoA method has become a hot topic in indoor positioning system (IPS).

**Figure 14:**
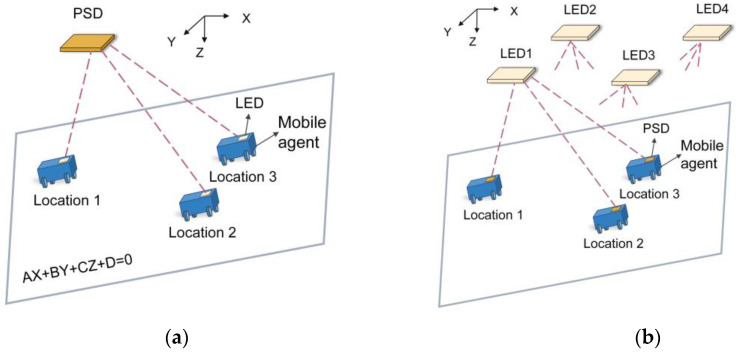
Schematic diagram of the posture measurement system based on the AoA method (**a**) 3D spatial positioning using a single RLED; (**b**) 6-DOF posture measurement using four RLEDs.

David et al. presented an AoA-based IPS by using a single PSD [69]. The system configuration is shown in Figure 14a. The receiver (PSD + lens) is fixed to a known position on the ceiling. An InfraRed Emitter Diode (IRED) is attached to the target which moves in a plane. Firstly, electrical calibration and geometric calibration must be carried out. Electrical calibration is to correct the errors which would affect the determination of the incident points. Then, the geometric calibration is to obtain the intrinsic parameters of the system, which would be used in the calculation of angle of arrivals. A detailed calibration process is illustrated in the authors’ previous work [70,71]. Once the calibrations have been performed, the next step is to determine the equation of the motion plane (AX+BY+CZ+D=0) and the value of AoA. The angle of arrival $\theta_{x}$ and $\theta_{y}$ with respect to the PSD can be expressed as:

(6) $$\theta_{x}=\arctan\left(x/f\right),\theta_{y}=\arctan\left(y/f\right)$$

where $\left(x,y\right)$ are PSD reading, f is the focal length. Finally, the 3D coordinates of the mobile target are derived as:

(7) $$X=Ztan\theta_{x}\;,\;Y=Ztan\theta_{y}\;,Z=-D/\left(Atan\theta_{x}+Btan\theta_{y}+C\right)$$

To verify the proposed measurement method, static and dynamic experiments have been carried out. In the static experiment, the positioning accuracy was 3.43 mm in a workspace of 2.5 m height covering an area of 1.3 × 1.3 m^2^. In the dynamic experiment with a working height of 1 m, the positioning accuracy of the target with moving speed of 0.2 m/s could reach 0.49 mm. The position update frequency was 100 Hz, which exceeded the operating speed of CCD-based positioning systems with equivalent accuracy.

The advantages of the proposed system are cheapness, ease of installation, low computational load and high measurement speed. However, the application scenario for this system is relatively simple since the target is constrained to move on a plane. Such a system cannot be used in more demanding applications such as Unmanned Aerial Vehicle (UAV) localization. In fact, increasing the number of emitters not only makes it possible to locate a target moving arbitrarily in space, but it also allows the acquisition of the 3D orientation of the target.

By increasing the number of emitters to four, Alvaro et al. achieves 3D full pose (3D position and 3D orientation) detection of the target without any prior knowledge of the motion space [72,73]. The scheme of the proposed system is shown in Figure 14b. Four LED emitters are fixed to the ceiling in a specific arrangement and their locations are known in advance. The receiver (PSD + lens) is fixed on a mobile target. By adopting the Frequency-Division Multiple-Access (FDMA) scheme, the light beams emitted from four LEDs are modulated at 6, 8, 10 and 14 kHz, respectively, and simultaneously detected by a single PSD. The advantage of the FDMA strategy is that the interference between the emitters can be eliminated and the ambient light is effectively filtered out. The four photocurrents output from PSD are amplified, filtered and digitized into sequences, and then processed by in-phase and Quadrature(I/Q) demodulation to derive 2D coordinates of the four projection points. These solved projection points are used to calculate the full 3D pose based on the AoA principle and computer vision strategy. The experimental tests showed that the mean positioning error of proposed system was 48.7 mm and the mean angle error of x-axis, y-axis and z-axis were better than 0.32°, 0.35° and 0.16°, respectively within an effective working space of 2.47 m height covering an area of 2.2 m^2^.

Although the 3D positioning accuracy is lower (48.7 mm vs. 0.49 mm) compared to the former case, the proposed system does not need any prior information of the motion space and the target can move arbitrarily, such accuracy is still outstanding compared to the conventional IPS system which has positioning accuracies ranging from a few tens of centimeters to one meter. Since the emitters are mounted on the ceiling, it is possible to make full use of existing illumination facility for positioning. Such a visible light positioning (VLP) system has been regarded as a promising candidate for the next generation of IPS.

## 4. Discussion

### 4.1. Comparative Analysis of Research Works

PSDs have been widely acknowledged by researchers as the most attractive detectors in 6-DOF posture measurement, for it enables the systems with high resolution, high accuracy and rapid speed. In the field of large-scale equipment assembly, PSDs are superior to QDs for realizing large FOV and high stable laser tracking, thus improving the dynamic range and repeatability of 6-DOF measurement. Additionally, PSDs are much more rapid than CCDs and free the binocular vision method from the complex image processing tasks, thus increasing the 6-DOF measurement speed. In the field of calibrating the geometric motion errors of ultra-precision linear stage, PSDs significantly enhance the accuracy and resolution of 6-DOF measurement. The greatest accuracy reported is 0.8 μm for translation and 0.7″ for rotation, and the highest resolution reported is 0.02 μm for translation and 0.03″ for rotation. In the field of other emerging areas, such as mMT, MAIS and IPS, PSDs are used as building blocks for constructing customized 6-DOF posture measurement systems, providing a range of optional features such as high compactness, large dynamic ranges and fast speed. 

Table 3 compared different literatures in terms of performance indicators. It is obvious that the laser tracking method and binocular vision method are appropriate if one intends to conduct large-range pose measurement. The laser diffraction method and laser collimation methods are superior for high-accuracy and high-resolution pose measurement. The kinematic method and angle-of-arrival method should be considered if one prefers highly compact pose measurement systems.

### 4.2. Challenges and Future Directions

1. Based on the analysis results of the literature mentioned above, PSD-based 6-DOF posture measurement systems still need further exploration and improvement, which are mainly manifested in the following three aspects: Non-linear response: PSD has a certain degree of non-linearity, which is caused by non-desirable factors such as inhomogeneous resistive layers, non-zero load resistance and imperfect device isolation. The non-linearity is greater at the edges of photosensitive surface, which will reduce the accuracy of kinematic analysis method and binocular vision method since these two methods both use the edge area of PSDs.
2. Dark current increases with PSD area: There is still a lack of large-area PSDs with low dark currents, posing the challenge that large dynamic range and high accuracy are difficult to obtain simultaneously for all methods.
3. No internal gain: When detecting the weak light or working in long distance, the SNR will be not high enough to ensure high accuracy because PSD has no internal gains. Therefore, the working distances of laser diffraction method and laser collimation method are often sacrificed to obtain ultra-high resolution and extreme accuracy.

With the increasing improvement of semiconductor technology and the introduction of new materials, novel PSDs with large active area, low dark current and high internal gain will be designed and constructed. In addition, the development of error compensation technology, such as artificial neural networks, will effectively improve the PSD linearity. Therefore, the future PSD-based 6-DOF posture measurement system will develop towards higher measurement accuracy, larger measurement range and faster response speed, and it will play an increasingly important role in a wider range of fields, better meeting the actual requirement of different areas.

## 5. Conclusions

With the strong application demand, 6-DOF posture measurement has attracted great interests among the academic and industrial communities. As a high-speed non-contact sensor with ultra-high resolution and precision, PSD has become the most competitive candidate for high-performance 6-DOF posture measurement systems, which has been reported in a large number of studies. In this review, we analyzed the existing challenges faced by 6-DOF posture measurement systems and explained how PSDs can overcome these challenges. Then we summarized the research progress of PSD-based 6-DOF posture measurement technology in some cutting-edge applications, including large-scale equipment assembly, ultra-precision manufacturing and other emerging areas. We discussed the advantages and limitations of PSD methods and comprehensively compared different researches on PSD-based 6-DOF measurement technologies. Finally, we analyzed the further challenges developing directions. It is convenient for researchers to understand the research progress and find ideas to establish improved systems in the posture measurement filed.

## Figures and Tables

**Table 1:** Application areas of 6-DOF posture measurement.

Applications	Brief Description	Required Accuracy	Required Range
On-orbit Servicing [8]	Detecting the pose of the target spacecraft for capture, repair, and docking.	5 mm 2°	± 30 m ± 40°
On-site Assembly of Large-scale Components [9]	Detecting the posture of large components to guarantee assembly accuracy.	1 mm 0.25°	0~20 m 20°
Microgravity Active Vibration Isolation System [10]	Detecting the pose of lab bench to provide microgravity for scientific experiments.	0.5 mm 0.5°	± 10 mm ± 2.5°
Astronomical Observations [11]	Detecting the pose of secondary mirrors to improve the imaging quality of the telescope.	10 μm 1″	± 2 mm ± 1500″
Ultraprecision Manufacturing [12]	Detecting the motion errors of the linear stage to improve the manufacture accuracy.	0.5 μm 0.5″	± 100 μm ± 100″

**Table 3:** Summary of 6-DOF posture measurement methods using PSDs.

Authors	Method	Resolution	Accuracy	Repeatability	Range	Speed	Compactness
Dong [44] (2016)	Laser tracking	★★★★	★★★★	★★★★★	★★★★	★★★	★★★
Liu [49] (2022)	Binocular vision	★★	★★	★★★	★★★★★	★★★	★★★★★
Wang [38] (2020)	Binocular vision	★★★	★★★	★★	★★★★★	★★	★★★★★
Cheng [54] (2022)	Binocular vision	★★★	★★★	★★★	★★★	★★	★★★★★
Kim [56] (2000)	Laser diffraction	★★★	★★★★	★★★★	★★	★★★★	★★★
Kim [57] (2002)	Laser diffraction	★★★	★★★★★	★★★★	★★	★★★★	★★★
Lee [58] (2011)	Laser diffraction	★★★★★	★★★★	★★★★	★★	★★★★	★★
Feng [60] (2013)	Laser collimation	★★★★★	★★★★	★★★	★★	★★★★	★★★
Zhao [61] (2017)	Laser collimation	★★★★★	★★★★★	★★★	★★	★★★	★★★
Ren [62] (2020)	Laser collimation	★★★★★	★★★★★	★★★	★★★	★★★★	★★
Wang [64] (2008)	Kinematic analysis	★★★★★	★★★★	★★★	★★★	★★★★	★★★★
Gao [10] (2019)	Kinematic analysis	★★★	★★★	★★★	★★★★	★★★★	★★★★★
David [69] (2017)	Angle-of-arrival	★★	★★	★★	★★★★	★★★★	★★★★★
Alvaro [72] (2020)	Angle-of-arrival	★★	★★	★★	★★★★	★★★★	★★★★★

## Data Availability

Not applicable.
